# Billing fees for various common allergy tests vary widely across Canada

**DOI:** 10.1186/s13223-020-00426-0

**Published:** 2020-04-22

**Authors:** Jennifer Lisa Penner Protudjer, Lianne Soller, Elissa Michelle Abrams, Edmond S. Chan

**Affiliations:** 1 Department of Pediatrics and Child Health, The University of Manitoba, 501G-715 McDermot Avenue, Winnipeg, MB R3E 3P4 Canada; 2 George and Fay Yee Centre for Healthcare Innovation, Winnipeg, Canada; 3 The Children’s Health Research Institute of Manitoba, Winnipeg, Canada; 4 Institute of Environmental Medicine, Karolinska Institutet, Stockholm, Sweden; 5 Centre for Allergy Research, Karolinska Institutet, Stockholm, Sweden; 6 Food and Human Nutritional Sciences, The University of Manitoba, Winnipeg, Canada; 7 Division of Allergy & Immunology, Department of Pediatrics, Faculty of Medicine, The University of British Columbia, Vancouver, BC Canada; 8 BC Children’s Hospital Research Institute, Vancouver, BC Canada

**Keywords:** Billing costs, Food allergy, Health Economics, Oral immunotherapy

## Abstract

**Background:**

The prevalence of food allergy in Canada is high and has increased over time. To date, there are no Canadian data on the healthcare costs of visits to allergists.

**Methods:**

We sent an anonymous survey to allergist members of the Canadian Society of Allergy and Clinical Immunology (CSACI) between October and December 2019. Survey questions included demographic information and billing fees for various types of allergy visits and diagnostic testing.

**Results:**

Of 200 allergists who are members of CSACI, 43 allergists responded (21.5% response rate). Billing fees varied widely. The greatest ranges were noted for oral immunotherapy (OIT; both initial consultation [mean $198.70; range $0 to $575] and follow up/build up visits [mean $125.74; range: $0 to $575]). There were significant provincial differences in billing fees, as well as significant billing fee differences between hospital versus community allergists (e.g. oral food challenge [OFC]: $256.38 vs. $134.94, p < 0.01). Billing fees were higher outside of Ontario, with the exception of specific Immunoglubulin E (sIgE) testing and OIT visits.

**Conclusions:**

Greater standardization of billing fees across provinces and between hospital versus community allergy could result in more consistency of billing fees for OFC and OIT across Canada. Further knowledge of exact costs will help inform practice and policy in the diagnosis and management of food allergy.

## Background

The prevalence of food allergy is at an all-time high [1], and impacts household costs [2], by age but not disease severity [3]. In one study, families of children and adolescents with food allergy reported excess annual household costs of approximately $6200 Canadian (CAD) and $7500 CAD, respectively, compared to families without food allergy. Families living with food allergy also report poor quality of life [4–9] and food allergy-related bullying [10]. Healthcare related costs have also been described [11]. Data from the United States indicate that the annual cost of food allergy to the healthcare system is approximately $4.3 billion [11]. With an estimated self-reported prevalence of 7–8%, the rates of food allergy are comparable between the United States [12] and Canada [13, 14]. Owing to differences in healthcare systems, cost comparisons between Canada and elsewhere are challenging for many reasons. For example, unlike in the United States, access to healthcare and medication in Canada is generally not dependent on private insurance. Thus, the American cost estimates reported by Gupta et al. [11] are unlikely to reflect Canadian healthcare costs.

At present, there are no published Canadian data on the cost of food allergy to the healthcare system, particularly the cost of healthcare visits to allergists. Moreover, the Canadian Institute for Health Information (CIHI) does not report on billing fees for allergists who treat patients with food allergy. Rather, there are only published data on the number of emergency department visits for anaphylaxis and allergy [15]. But, the majority of patients with food allergy do not routinely—or ever—seek emergency care [16, 17]. As such, the ability to estimate non-emergency-related healthcare costs for food allergy is a necessary first step in establishing the costs of food allergy to the Canadian healthcare system. To this end, we aimed to provide a benchmark for government-paid reimbursement for common allergy tests amongst physicians, and for provinces looking at adjusting service fees.

## Methods

This anonymous survey was open between October and December 2019 to allergists who see children and/or adults for food allergy-related concerns. Allergists were recruited through the Canadian Society of Allergy and Clinical Immunology (CSACI) database, which includes the majority of Canadian allergists (approximately 200).

Potential participants received an introductory email, which contained a brief summary of the study and study team. If participants agreed to continue to the questionnaire, consent was assumed. We did not collect names or contact details of participants.

The questionnaire included demographic questions (province, hospital or community practice) and billing fees for various food allergy-related visits: initial diagnosis of food allergy, follow up visit for food allergy, oral food challenge (OFC), initial visit for oral immunotherapy (OIT), OIT build up visit, skin prick test, and ImmunoCap test (specific Immunoglobulin [sIgE] blood test). The questionnaire was created in Survey Monkey©, with results aggregated and sent to the first author.

Location of practice was self-reported as either community or hospital. Provincial level data were provided for each type of food allergy-related visit. No allergists are known to have a primary practice in any of the three Canadian territories (Yukon, Northwest Territories, Nunavut).

### Statistical analysis

If an allergist provided a cost range for any of the queried services (initial diagnosis; follow up visit; OFC; initial visit for OIT; OIT build up visit, SPT; sIgE, the mean of the range was used. If the allergist parceled out costs based on billing party (i.e. allergist vs. hospital), these amounts were summed to ascertain the full billing amount. If an allergist reported that the costs were covered by his/her respective provincial health authority, and/or noted that these costs were comparable to other forms of consults, and/or gave a cost per hour, and/or selected “don’t know” and/or did not provide any quantitative data, their results were excluded from the analysis.

Data were analysed using descriptive statistics (n/N, %, mean, standard deviation, range, t-tests, Mann–Whitney U-tests). A *p* value < 0.05 was considered statistically significant. Data were analysed using Stata 15.1 (College Station, TX). Analyses were performed on the entire data set. Subsequently, billing fees were compared between community vs. hospital allergists. Then, comparisons of billing fees were made between provinces, with the most commonly reported province (Ontario), as the reference location. Finally, we made comparisons between community vs. hospital allergists in provinces where sample sizes were sufficient for robust analyses. This study was approved by The University of Manitoba Health Research Ethics Board HS23227 (H2019:367).

## Results

Of approximately 200 allergist members of CSACI, 43 allergists participated in this survey (response rate = 21.5%). Most responding allergists (30/43; 69.8%) reported a community practice location (Table 1). Nearly half of allergists reported practicing in Ontario (19/43; 44.2%). Fewer allergists reported a location of practice in Quebec (10/43; 23.3%) or provinces in Western Canada (British Columbia 5/43; 11.6%; Alberta 4/43; 9.3%; Saskatchewan 0/43; 0%; Manitoba 2/32; 4.7%). Few allergists from Atlantic Canada participated (Nova Scotia 2/43; 4.7%; Newfoundland and Labrador 1/42; 2.3%).

**Table 1:** Demographic characteristics of participating allergists (N = 43)

	N	%
Practice location		
Community	30	69.8
Hospital	13	30.2
Geographic location of practice		
British Columbia	5	11.6
Alberta	4	9.3
Saskatchewan	0	0.0
Manitoba	2	4.7
Ontario	19	44.2
Quebec	10	23.3
Nova Scotia	2	4.7
Prince Edward Island	0	0.0
New Brunswick	0	0.0
Newfoundland and Labrador	1	2.3

For all common allergy tests, billing fees varied widely. The mean billing fee for an initial visit for food allergy was $205.60, but ranged from $122 to $500 (Table 2). Of the allergists reporting initial visit fees greater than the mean, 76.9% (10/13) practiced in Western Canada.

**Table 2:** Mean billing fees (CAD) for common allergy visits and tests (N = 43)

	All responding allergists			
	N	Mean	Standard deviation	Range
Initial visit	42	205.60	73.29	122–500
Follow up visit	42	88.66	45.60	40–300
Oral immunotherapy				
Initial visit	34	198.79	153.15	0–625
Build up/follow up	33	125.74	146.08	0–575
Oral food challenge	36	172.05	132.61	4.60–600
Skin prick testing	42	11.82	23.88	0.08–110
Blood testing (sIgE)	35	13.40	14.10	0–40

**Fig. 1:**
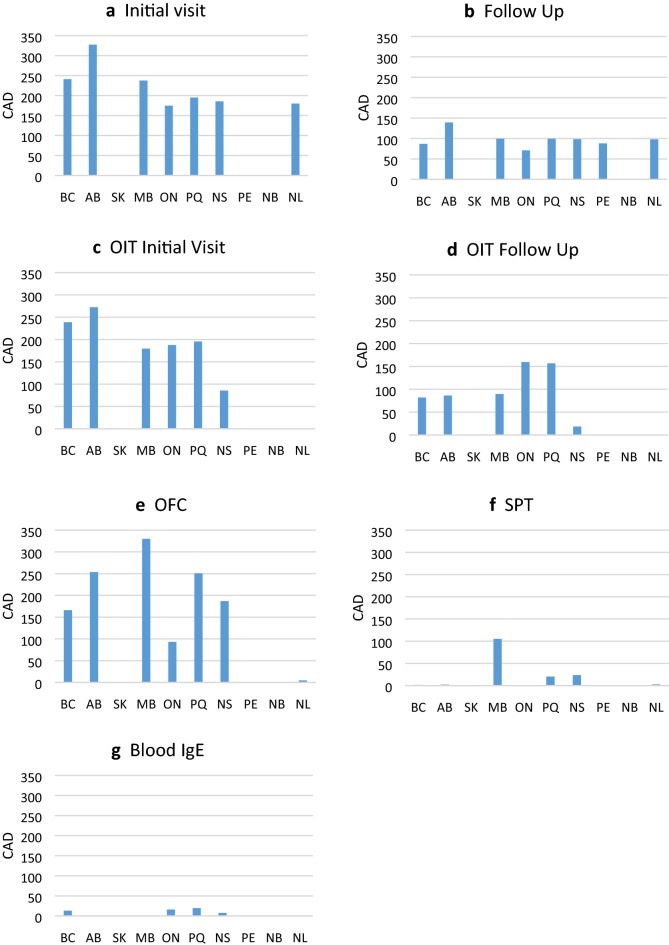
Billing fees (CAD) for common allergy visits and tests, by province

The greatest ranges for all billing fees were noted for OIT, where an initial visit was charged at a mean of $198.70, but ranged from $0 to $625 (Fig. 1c). Of the allergists reporting initial OIT visit fees greater than the mean, 34.8% (8/23) practiced in Ontario and 21.7% (5/23) practiced in Quebec.

For a build up/follow up OIT visit, the mean billing fee was $125.74, but ranged from $0 to $575 (Fig. 1d). Of the allergists reporting build up/follow up OIT visit fees greater than the mean, 47.6% (10/21) practiced in Ontario and 38.1% (8/21) practiced in Quebec.

The narrowest range of billing fees was reported for sIgE testing, with a mean of $13.40, and a range of $0-$40 (Fig. 1g). Excluding all reported billing fees of $0 did not dramatically alter the mean of the various billing fees. With these exclusions, the corresponding numbers were, OIT initial visit: mean $241, range $59 to $ 625; OIT build up/follow up: mean $159, range $10 to $575; blood (sIgE) testing: mean $25, range $10 to $40.

Compared to community allergists, hospital allergists had higher billing fees for OFC ($134.94 vs. $256.38, p < 0.01), but not other common allergy tests such as initial or follow up visits, visits related to OIT, or SPT or sIgE testing.

Compared to Ontario, mean billing fees in other parts of Canada were significantly higher for initial and follow up visits for food allergy ($174.65 vs. $228.82, p < 0.01; $71.00 vs. $101.90, p < 0.001, respectively), OFC ($93.21 vs. $222.22, p = 0.03), skin prick tests ($1.05 vs. $19.90, p < 0.01); but not for OIT initial visits or build up/follow up visits, or blood sIgE testing (all p > 0.05).

Comparisons of billing fees between community and hospital-based allergists within a province were possible in Ontario (n = 16, n = 3, respectively) and Quebec (n = 3; n = 7, respectively), but not other provinces due to small sample size. In these two provinces, billing fees did not differ (all p > 0.05) by location of practice. Of note, no Quebec-based community allergists reported billing fees for follow up visits for OIT.

## Discussion

In this survey of Canadian allergists, the reported billing fees for various common allergy tests varied widely, with widest ranges noted for initial OIT visits and build up/follow up OIT visits. At a national level, billing fees were comparable between community vs. hospital allergists with the exception of billing fees for OFC, which were significantly higher amongst hospital allergists. At a provincial level, differences were attenuated, which could be due to small sample sizes. Billing fees were higher in other parts of Canada compared to Ontario, with the exception of OIT initial/follow-up visits and sIgE testing.

Interestingly, fewer allergists responded to questions on OIT billing fees than other common allergy tests. Although it would be incorrect to assume based on this finding alone that not all allergists who provide consultation for food allergy offer OIT, roughly 34 allergists providing initial OIT visits out of 42 allergists providing initial food allergy visits (81%) is higher than that of an American report in which only about 14% of allergists reported using OIT [18]. We also acknowledge that Canadian provinces have not yet introduced billing fee codes for OIT, which may explain why some allergists reported a fee of $0 for this procedure (i.e. $0 may have represented the government billing fee amount while not capturing the privately billed amount.)

Overall, hospital allergists reported higher billing fees for OFC than community allergists, although these differences are not significant in Ontario and Quebec, the provinces for which sample size permitted comparison, but which may nonetheless be too small to detect any differences. OFCs are the gold standard to establish a diagnosis of food allergy or the development of tolerance. Importantly, it is possible that community allergists may refer to hospital-based allergists for higher risk challenges, whereas those completed in the community may be performed to confirm tolerance. This practice aligns with recommendations from the United States, in which sIgE and SPT have been shown to be useful in determining the most appropriate place to perform an OFC [19]. At the same time, we recently reported that many allergists view a lack of resources, inconsistent standards between hospital and community practices [20], and a lack of comfort [21] as barriers to performing OFCs. These barriers warrant consideration going forward, as does a comprehensive cost-effectiveness analysis of OIT.

Billing fees in Ontario were significantly lower for initial and follow up visits for food allergy than in other Canadian provinces. This may be partly explained by a tendency for more private billing in Ontario than other parts of Canada.

We acknowledge the limitations of our study, including a response rate of 21.5%. However, this rate parallels the response rate in a 2018 survey of CSACI members of 22% [21], and highlights an overall decreasing temporal trend in response rates in recent years [20–22]. As a result of this low response rate, sample sizes were low. For provinces other than Ontario and Quebec, we were not able to compare billing fees at a provincial level. Lastly, our study did not distinguish between billing fees paid to allergists by their provincial government versus fees paid out-of-pocket by patients. Likewise, we did not identify the province(s) in which fees were paid out-of-pocket although it is common knowledge that Ontario labs outside of hospitals charge patients privately for sIgE testing and some Ontario allergists charge privately for OIT [23].

We highlight that this is the first Canadian study to estimate billing fees for common allergy visits and tests. These estimates are essential to better understand the health economic impact of food allergy, and should be used in combination with healthcare register data to calculate non-emergent allergy-related costs. Moreover, these estimates will form part of further economic analyses by our group of the cost burden of OIT, and may inform other cost analyses, including comparisons between common allergy tests. The prevalence of food allergy is at an all-time high, and disproportionately affects children, including many who are unlikely to outgrow their allergies [1]. Thus, accurate estimates of the cost burden beyond emergency department visits for allergy and anaphylaxis are critical for healthcare planning and policy in Canada.

## Conclusions

Billing fees for various common allergy visits and tests vary widely across Canada, with the widest ranges reported for OIT. Overall, hospital allergists report higher billing fees for OFC than community allergists. Such differences were attenuated in provincial level comparisons. Billing fees are higher outside of Ontario, with the exception of OIT visits and sIgE testing. These data provide a benchmark for reimbursement for OIT amongst physicians, as well as for provinces looking to adjust billing fees. Greater standardization of billing fees across provinces and between hospital versus community allergy could result in more consistency of billing fees for OFC and OIT across Canada. Further knowledge of exact costs will help inform practice and policy in the management of food allergy.

## Data Availability

The datasets analysed in the current study are not publicly available due to the real potential of identifying individual allergists in regions with few practicing allergists. EMA is a member of the healthcare advisory board for Food Allergy Canada. ESC has received research support from DBV Technologies, has been a member of advisory boards for Pfizer, Pediapharm, Leo Pharma, and Kaleo, is a member of the healthcare advisory board for Food Allergy Canada, was an expert panel and coordinating committee member of the National Institute of Allergy and Infectious Diseases (NIAID)-sponsored Guidelines for Peanut Allergy Prevention, and is co-lead of the CSACI oral immunotherapy guidelines.

## Abbreviations

CAD: Canadian Dollars
CIHI: Canadian Institute for Health Information
CSACI: Canadian Society of Allergy and Clinical Immunology
sIgE: Specific Immunoglobulin E
OFC: Oral Food Challenge
OIT: Oral immunotherapy
